# Evaluating Large Language Models for Mild Cognitive Impairment: A Bilingual Comparison of ChatGPT, Gemini, and Kimi

**DOI:** 10.1093/geroni/igaf122.2247

**Published:** 2025-12-31

**Authors:** Yexuan Xiao, Qianhui Pan, Nan Jiang, Haoyuan Liu, Yilin He, Yuhe Zhang, Tingmei Wang

**Affiliations:** Tsinghua University, Beijing, Beijing, China (People’s Republic); Tsinghua University, Beijing, Beijing, China (People’s Republic); Tsinghua University, Beijing, Beijing, China (People’s Republic); Tsinghua University, Beijing, Beijing, China (People’s Republic); Tsinghua University, Beijing, Beijing, China (People’s Republic); Tsinghua University, Beijing, Beijing, China (People’s Republic); Tsinghua University, Beijing, Beijing, China (People’s Republic)

## Abstract

**Background:**

Mild Cognitive Impairment (MCI) is a key stage between normal aging and Alzheimer’s Disease (AD), with early intervention crucial for slowing progression. Large Language Models (LLMs) offer promising support by providing accessible, evidence-based information for non-specialist healthcare professionals and care partners. However, concerns about accuracy and limited multilingual evaluations remain.

**Objective:**

This study explores the potential of LLMs in managing MCI, examines their support for non-specialist healthcare professionals and care partners, and compares English and Chinese responses to MCI-related queries, considering language-specific nuances and effectiveness.

**Methods:**

We submitted 72 open-ended questions related to MCI management to ChatGPT-4o, Gemini, and Kimi, assessing their responses based on accuracy, comprehensibility, specificity, and actionability using a five-point Likert scale. Statistical analyses, including Intraclass Correlation Coefficients and Mann-Whitney U tests, were conducted to examine response across models.

**Result:**

LLMs’ performance in MCI management was evaluated, with the Symptoms and Diagnosis domain scoring highest. Healthcare professionals’ needs were better met than care partners’, particularly in accuracy, comprehensibility, and actionability. English responses outperformed Chinese in comprehensibility and specificity.

**Conclusion:**

Based on the results, LLMs demonstrate potential in assisting non-specialist healthcare professionals and care partners, particularly in the domains of symptoms and diagnosis. However, there is a need for further optimization in Chinese medical corpora, as English responses outperformed Chinese ones due to corpus disparities. Tailored models for care partners, focusing on reliability and clarity, are essential to address their unmet needs and enhance their experience in managing MCI.

